# Innovative Composite Materials for Sound Absorption and Insulation: Where We Are and Where We Are Going

**DOI:** 10.3390/ma14081954

**Published:** 2021-04-14

**Authors:** Francesco Martellotta

**Affiliations:** Dipartimento di Scienze dell’Ingegneria Civile e dell’Architettura, Politecnico di Bari, Via Orabona, 4, 70125 Bari, Italy; francesco.martellotta@poliba.it; Tel.: +39-080-5963631

Materials with sound-absorbing or sound-insulating properties have been rapidly evolving in recent years due to several reasons. On one side, there is the ever-increasing awareness of the adverse effects that noise and lack of acoustic comfort may have on human health. On the other, the availability of more sophisticated fabrication techniques, calculation methods, and new materials, has stimulated researchers and, more and more frequently, industry to develop customized materials with improved properties.

For sound-absorbing materials, combining a sufficiently wide frequency range of action with reduced mass and thickness, as well as with aesthetic requirements (due to the need to use such materials in spaces like classrooms, restaurants, train stations, etc.), is becoming essential, not to mention durability and ease of maintenance, which should always be considered. For sound-insulating materials, aesthetic problems are typically not an issue, but other aspects, such as balancing low dynamic stiffness with load-bearing properties, become important in order to ensure the best performance. In addition, whatever the purpose, such devices should be sustainable, have a low impact in terms of life cycle assessment, and possibly involve the use of recycled materials or natural products.

All of the above questions clearly represent a challenge for researchers, but at the same time, they offer new opportunities to experiment with cutting-edge solutions, like those based on the use of composite materials, as well as including nanotechnologies, “green” vegetal and animal fibers, and metal/ceramic/polymer matrixes, up to the use of acoustic metamaterials.

In this Special Issue of *Materials* [1], all of these topics are covered, providing an interesting picture of the current trends. The potential of perforated panels, that, thanks to 3D printing and refined numerically controlled manufacturing processes may now offer flexibility of use, was investigated by Li et al. [2] to obtain improved low-frequency performance thanks to a parallel arrangement of four different perforated panel absorbers in which the number and dimensions of apertures ensure a smooth manufacturing process compared to micro-perforated panels. The analytical models available to study such a group of materials also showed very good accuracy in predicting actual behavior, thus representing a further element in favor of such applications. A parallel arrangement of perforated plates with “extended” tubes was also investigated with reference to their diffuse field behavior [3]. A significant dependence of sound absorption as a function of incidence angle was found, and as a function of the period absorber layout, which made sound absorption decrease when the period was comparable with the wavelength. However, proper design ensured good sound absorption in the mid–low frequency range when using such a parallel arrangement of perforated absorbers.

The potential offered by additive manufacturing techniques in terms of customization of the porous cell structure of materials aimed at obtaining broad-band sound absorption was investigated by Cavalieri et al. [4]. They showed that, at least in theory, given the current limitations of the fabrication techniques, it could be possible to obtain a significantly high and broad-band sound absorption by using numerical optimization of geometrical properties of porous cells. Investigation on the role of grading properties and anisotropy showed that they can contribute to further maximizing absorption. Similarly, the role of the geometrical and microstructural properties of granular molecular sieves (i.e., zeolite crystals) was experimentally investigated by Zhou et al. [5]. They found that the intrinsic porosity of the granules is less important than the actual grain size and overall thickness of the layer in affecting sound absorption. In any case, a good fit with phenomenological model predictions was found, allowing for possible optimization of the material properties.

A second group of papers focused its attention on the use of natural or recycled components to create a skeleton of porous materials with sound-absorbing purposes. Cigarette butts of different lengths and under different conditions (smoked or non-smoked) were used by Gómez Escobar et al. [6] to produce small samples that were tested in a standing wave tube. The results showed that acceptable sound absorption could be obtained from about 2 kHz on, with small variations depending on the butts’ length and smoking conditions. Rubino et al. [7] investigated the potential of recycled textile waste to produce composite sound-absorbing panels in combination with bio-based binders like chitosan and gum Arabic. Sound absorption coefficients, flow resistivity, and other physical properties were determined and the results showed relatively little influence of the binder on the acoustic behavior. Conversely, sample density and flow resistivity played a major role in changing the frequency dependence of sound absorption. The use of natural materials as the main component of granular porous sound absorbers was also the topic of research from Liuzzi et al. [8]. In this case, almond skins were dried and then mixed with different binders (including gum Arabic and polyvinyl acetate glue) to obtain, after further drying, a rigid panel. The acoustic properties in this case were enhanced by the very complex microstructure that originates from the mixture, resulting in higher absorption coefficients appearing at lower frequencies than for other absorbing materials of similar thickness. The use of environmentally friendly fire retardants was investigated by Liu and Hu [9] with reference to polyurethane foams, which are well known for their sound-absorbing properties, in combination with phase change materials.

Fediuk et al. [10] reviewed the current literature on the topic of innovative concretes with acoustic-oriented properties. Thus, treatments oriented to improve both sound-absorbing and sound-insulating characteristics were discussed, including porous and aerated concretes, concretes with special aggregates like recycled rubber crumbs, expanded polystyrene, synthetic fibers, recycled aggregates, mollusk shells, and foam glass. Detailed case studies of building components are provided.

The last two papers of the Special Issue specifically address the topic of sound insulation. Santoni et al. [11] investigated the use of wood flour as filler of wood plastic composite (WPC) panels. WPC boards are analyzed both numerically and experimentally with reference to the vibrational and transmission loss behavior of such panels. Junctions and boundary conditions proved to significantly affect the results, pointing out the importance of a proper design and modeling of such devices. Finally, Ehrig et al. [12] investigated the potential of compressible constrained layer damping (CCLD) that is a semi-active, lightweight-compatible solution for vibration mitigation based on a base structure, a constraining plate, and a compressible open-cell foam core in between, enabling the adjustment of the structure’s vibration behavior by changing the core compression using different actuation pressures. The results showed that such changes affected transmission loss in the range between 1 kHz and 5 kHz, with the best performance appearing when maximum compression was applied.

Overall, a challenging and promising scenario is depicted, suggesting that in the coming years, substantial innovations might move from research labs to the market, actually contributing to improved performance combined with a lower environmental impact.
